# Impacts of gold nanoparticles on MHD mixed convection Poiseuille flow of nanofluid passing through a porous medium in the presence of thermal radiation, thermal diffusion and chemical reaction

**DOI:** 10.1007/s00521-016-2688-7

**Published:** 2016-11-22

**Authors:** Sidra Aman, Ilyas Khan, Zulkhibri Ismail, Mohd Zuki Salleh

**Affiliations:** 1 Futures and Trends Research Group, Faculty of Industrial Science and Technology, Universiti Malaysia Pahang (UMP), Lebuhraya Tun Razak, 26300 Kuantan, Pahang Malaysia; 2 Basic Engineering Sciences Department, College of Engineering Majmaah University, Majmaah, 11952 Saudi Arabia

**Keywords:** Gold nanoparticles, Mixed convection, Kerosene oil, Chemical reaction, Heat and mass transfer, MHD, Porous medium, Heat transfer rate

## Abstract

Impacts of gold nanoparticles on MHD Poiseuille flow of nanofluid in a porous medium are studied. Mixed convection is induced due to external pressure gradient and buoyancy force. Additional effects of thermal radiation, chemical reaction and thermal diffusion are also considered. Gold nanoparticles of cylindrical shape are considered in kerosene oil taken as conventional base fluid. However, for comparison, four other types of nanoparticles (silver, copper, alumina and magnetite) are also considered. The problem is modeled in terms of partial differential equations with suitable boundary conditions and then computed by perturbation technique. Exact expressions for velocity and temperature are obtained. Graphical results are mapped in order to tackle the physics of the embedded parameters. This study mainly focuses on gold nanoparticles; however, for the sake of comparison, four other types of nanoparticles namely silver, copper, alumina and magnetite are analyzed for the heat transfer rate. The obtained results show that metals have higher rate of heat transfer than metal oxides. Gold nanoparticles have the highest rate of heat transfer followed by alumina and magnetite. Porosity and magnetic field have opposite effects on velocity.

## Introduction

**Fig. 1:**
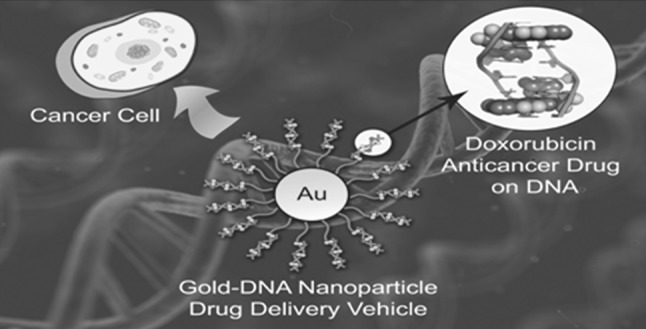
Application of gold nanoparticles in cancer therapy

Gold is one of the initial metals that have been originated. It has appealed the researchers owing to its unique properties and extended implementations. In technology, gold is used as an organic photovoltaic, drug delivery in nanotechnology (medical implementation) and catalysis. However, in the present study we concentrate on gold particles of nanosizes. Gold nanoparticles (AuNP) also named as colloidal, a suspension of nanometer-sized gold particles in a carrier fluid. They consist of a Au core and a surface coating. The evaluation of colloidal gold started with the work of Michael Faraday in 1850s [1]. Later on, in 1857, Faraday researched the optical characteristics of colloidal gold. He composed the fundamental sample of colloidal gold or (AuNP) which he specified as activated gold [2]. He observed that colloidal solution has possibly two colors (sharp red or yellowish) relying on its dimension [3, 4]).These properties are due to interaction with light studied by Jain et al. [5]. Hurst and Sarah [6] investigated that gold nanoparticles can be used in drug delivery. Their properties are adjustable by uttering the size, shape and surface chemistry. In addition to Au core, a protective coat which surrounds the core can also be modified to control particle stability and solubility. Sohyoung Her et al. [7] studied an experimental mechanism of gold nanoparticles for application in cancer radiotherapy. Doxorubicin/gold nanoparticles for cancer therapy through the enhanced tumor targeting were experimentally investigated by Kim et al. [8]. Lodice et al. [9] enhanced photo-thermal cancer therapy by gathering gold nanoparticles in form of nanostructure. Gold nanoparticles have been used in electronics as conductors in printable inks, electronic chips and resistors. In hyperthermia therapy, the particles heat up when light of wavelengths from 700 to 800 nm is applied to a tumor accommodating gold nanoparticles and kill up the targeted cells. [10–12] investigated gold nanoparticles as an agent for cancer therapy. Hainfeld et al. [13] used (AuNP) for the first time to amplify radiation dose. They introduced 1.9-nm (AuNP) into mice having cancer in the thighs and then expose the tumor to radiation 2 mints later. Recently, dose amplification in MDA-MB-231 breast cancer cell is discussed by Jain et al. [14]. Huang and El-Sayed [15] discussed implementations in cancer diagnosis and treatment. In Fig. 1, gold nanoparticles are shown with its applications in cancer therapy.

However, gold nanoparticles are rarely used for studying heat transfer rate due to mixed convection. Although mixed convection occurs in many industrial and technological processes such as chemical processing, food processing industry, nuclear reactors, electronics cooling technology and thermal insulations, the studies on gold nanoparticles in this direction are scarce. However, for other types of nanoparticles, enough literature has been developed. For example, Abu-Nada and Chamkha [16] studied mixed convection flow of a nanofluid. They considered lid-driven cavity along with wavy wall. Ajmera [17] investigated experimentally mixed convection in multiple ventilated enclosures with discrete heat source. [18–25] also reported similar studies.

In nanofluids, chemical reaction of nanoparticles with base fluid is required to take place in such problems to absorb the suspended particles within the base fluid. Various authors studied heat and mass transfer problems with chemical reaction. Among them, Kandasamy [26] studied impact of chemical reaction on Cu, ${\text{Al}}_{2} {\text{O}}_{3}$ and SWCNTs-nanofluid flow under slip conditions. Pal and Biswas [27] and Odat and Azab [28] used perturbation analysis to study magneto-hydrodynamics flows with chemical reactions.

In the present work, we have chosen gold nanoparticles due to its high thermal conductivity and adjustable surface chemistry. More exactly, this work is concentrated on MHD mixed convection Poiseuille flow of fluid with gold (AuNP) nanoparticles passing taking thermal radiation, thermal diffusion and chemical reaction into account with porosity. The problem is solved analytically impacts of cylindrical shape gold nanoparticles on MHD mixed convection Poiseuille flow of nanofluid passing through a porous medium under the influence of thermal radiation, thermal diffusion and chemical reaction. This research mainly focuses on gold nanoparticles; however, for the sake of comparison, four other types of nanoparticles namely silver, copper, alumina and magnetite are analyzed for the heat transfer rate. Analytical solutions are computed using the perturbation technique and discussed in various plots and tables. Although many researchers have done experimental work on gold nanoparticles, very less work has been done on this topic analytically.

## Formulation and solution of the problem

**Fig. 2:**
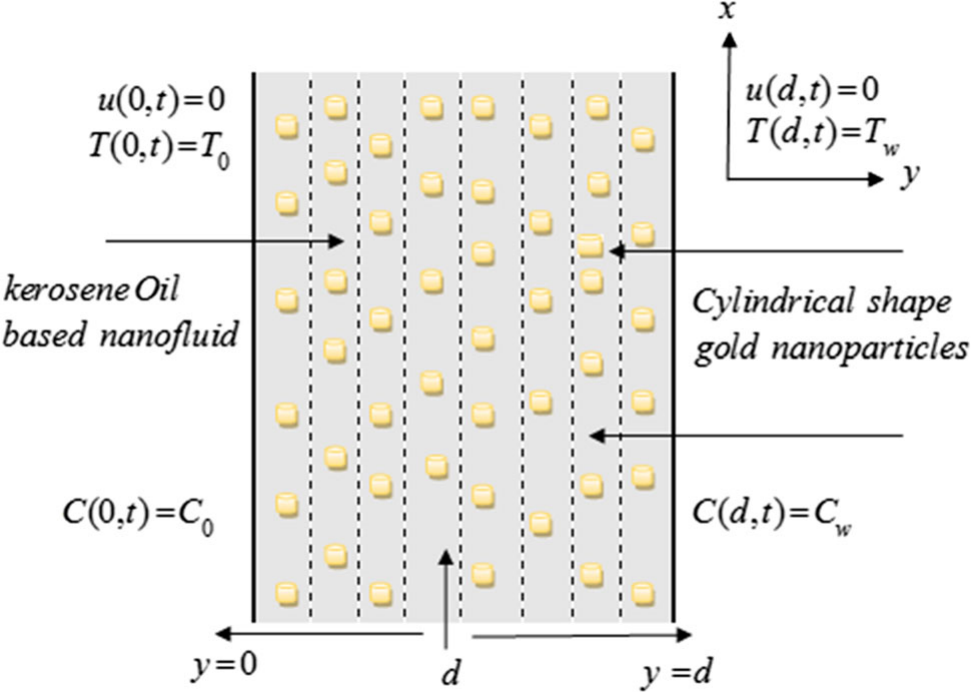
Poiseuille flow of nanofluid with gold nanoparticles

Consider MHD mixed convection flow of a nanofluid composed of gold nanoparticles (AuNP) suspended in kerosene oil taken as base fluid in a vertical channel with saturated porous medium in the presence of chemical reaction. Mixed convection is induced by buoyancy force and external pressure gradient. This fluid is made electrically conductor by applying a magnetic field perpendicular to the flow. Both plates temperature and concentration $T_{0} ,\,C_{0}$ and $T_{w} ,\,C_{w}$ are supposed to be high sufficiently and generate the radiative heat transfer as in Makinde and Mhone [29]. The physical geometry of the problem is shown in Fig. 2. Under the above assumptions, the problem is governed by the following set of partial differential equations:

1 $$\rho_{\text{nf}} \frac{\partial u}{\partial t} = - \frac{\partial p}{\partial x} + \mu_{\text{nf}} \frac{{\partial^{2} u}}{{\partial y^{2} }} - \left( {\sigma_{\text{nf}} B_{0}^{2} + \frac{{\mu_{\text{nf}} }}{{k_{1} }}} \right)u + \left( {\rho \beta_{T} } \right)_{\text{nf}} g\left( {T - T_{0} } \right) + \left( {\rho \beta_{c} } \right)_{\text{nf}} g\left( {C - C_{0} } \right),$$

2 $$(\rho c_{\rm p} )_{\text{nf}} \frac{\partial T}{\partial t} = k_{\text{nf}} \frac{{\partial^{2} T}}{{\partial y^{2} }} + 4\alpha_{0}^{2} \left( {T - T_{0} } \right),$$

3 $$\frac{\partial C}{\partial t} = D_{\text{nf}} \frac{{\partial^{2} C}}{{\partial y^{2} }} + \frac{{D_{\text{nf}} K_{T} }}{{T_{m} }}\frac{{\partial^{2} T}}{{\partial y^{2} }} - k_{\text{r}} \left( {C - C_{0} } \right),$$

with boundary conditions

4 $$\begin{array}{*{20}l} {u(0,t) = 0,} \hfill & {u(d,t) = 0,} \hfill \\ {T(0,t) = T_{0} ,} \hfill & {T(d,t) = T_{w} ,} \hfill \\ {C(0,t) = C_{0} ,} \hfill & {C(d,t) = C_{w} .} \hfill \\ \end{array}$$

Fluid velocity is in the *x*-direction, denoted by $u = u\left( {y,\,t} \right),$ $T = T\left( {y,\,t} \right)\,$ is the temperature, $\rho_{\text{nf}}$ signifies the density, the dynamic viscosity is symbolized by $\mu_{\text{nf}}$, $\sigma_{\text{nf}}$ is the electrical conductivity, the permeability of the porous medium is represented by $k_{1} > 0$, $\left( {\rho \beta } \right)_{\text{nf}}$ expresses the coefficient of thermal expansion, $g$ is the gravitational acceleration, $\;\left( {\rho c_{\rm p} } \right)_{\text{nf}}$ is the heat capacitance, the thermal conductivity $k_{\text{nf}}$ of nanofluids, $k_{\text{r}}$ is chemical reaction parameter, $\alpha_{0}^{{}}$ is the radiation absorption coefficient and $D_{\text{nf}}$ is thermal diffusivity. The subscript ${\text{nf}}$ corresponds to nanofluid. The pulsatile pressure gradient used by Hayat et al. [30] defined as $- \partial p/\partial x = \lambda_{0} + \lambda_{1} \varepsilon \exp \left( {i\omega t} \right),\,$ in the flow direction is used, where $\lambda_{0}$ and $\lambda_{1}$ are constant and $\omega$ signifies the frequency of oscillation.

Thermal conductivity and dynamic viscosity are defined by Hamilton and Crosser model [31], as this model can be used for both kinds of nano-particles, i.e., spherical and non-spherical; see for example Aaiza et al. [22]. This model is defined as:

5 $$\mu_{\text{nf}} = \mu_{\rm f} (1 + a\phi + \phi^{2} b),$$

6 $$\frac{{k_{\text{nf}} }}{{k_{\rm f} }} = \frac{{k_{\rm s} + (n - 1)k_{\rm f} + (n - 1)(k_{\rm s} - k)\phi }}{{k_{\rm s} + (n - 1)k_{\rm f} - (k_{\rm s} - k_{\rm f} )\phi }},$$

The density $\rho_{\text{nf}}$, coefficient of thermal expansion $\left( {\rho \beta } \right)_{\text{nf}} ,$ heat capacitance $\left( {\rho c_{\rm p} } \right)_{\text{nf}}$ and thermal conductivity $\sigma_{\text{nf}} ,$ of nanofluids are used by Aaiza et al. [22]:

7 $$\begin{array}{*{20}l} {\rho_{\text{nf}} = (1 - \phi )\rho_{\rm f} + \phi \rho_{\rm s} ,} \hfill & {\left( {\rho \beta } \right)_{\rm nf} = (1 - \phi )\left( {\rho \beta } \right)_{\rm f} + \phi \left( {\rho \beta } \right)_{\rm s} ,} \hfill \\ {\left( {\rho c_{\rm p} } \right)_{\rm nf} = (1 - \phi )(\rho c_{\rm p} )_{\rm f} + \phi (\rho c_{\rm p} )_{\rm s} ,} \hfill & {\sigma_{\rm nf}} = {\sigma_{\rm f}}\left[{1}+\frac{{3}(\frac{\sigma_{s}}{\sigma_{f}}-1)\phi}{(\frac{\sigma_{s}}{\sigma_{f}}+2)-(\frac{\sigma_{s}}{\sigma_{f}}-1)\phi}\right]\end{array}$$

where $\phi$ symbolizes the volume fraction, $\rho_{\text{f}}$ and $\rho_{\text{s}}$ indicate density of the carrier fluid and gold nanoparticles, $\beta_{\text{s}}$ and $\beta_{\text{f}}$ is the coefficient of thermal expansions, $(c_{\text{p}} )_{\rm s}$ and $(c_{\text{p}} )_{\text{f}}$ is the heat capacities at a certain pressure, $\beta_{T}$ is the thermal expansion coefficient and $\beta_{c}$ is the solutal expansion coefficient, a property of particle physique signified by, $a$ and $b$ specified in Table 1, Timofeeva et al. [32]. Thermophysical properties of base fluid and nanoparticles are given in Table 2 as mentioned by [22].

**Table 1:** Empirical shape factors

Model	Cylinder
*a*	13.5
*b*	904.4

**Table 2:** Thermophysical properties of kerosene oil and nanoparticles

Material	Symbol	$\rho$ (kg/m^3^)	$c_{\text{p}} ({\text{kg}}^{ - 1} \,{\text{k}}^{ - 1} )$	$k\,({\text{W}}/{\text{mk}})$
Gold	Au	19,300	129	318
Kerosene oil	–	783	2090	0.145
Silver	Ag	10,500	235	429
Magnetite	${\text{Fe}}_{3} {\text{O}}_{4}$	5180	670	9.7
Alumina	${\text{Al}}_{2} {\text{O}}_{3}$	3970	765	40
Copper	${\text{Cu}}$	8933	385	401

The empirical shape factor $n$ in Eq. (6) expresses as $n = 3/\varPsi ,\,\;$ where $\varPsi$ signifies sphericity of the particle. Its value for cylindrical physique particles is stated in Table 3 as used by Timofeeva et al. [32].

**Table 3:** Sphericity $\varPsi$ for various shapes nanoparticles

Model	Cylinder
$\varPsi$	0.62

Using the non-dimensional variables

8 $$\begin{aligned} & u^{ * } = \frac{u}{{U_{0} }},\quad x^{ * } = \frac{x}{d},\quad t^{ * } = \frac{{tU_{0} }}{d},\quad y^{ * } = \frac{y}{d},\quad p^{ * } = \frac{d}{{\mu U_{0} }}p, \\ & T^{ * } = \frac{{T - T_{0} }}{{T_{w} - T_{0} }},\quad C^{ * } = \frac{{C - C_{0} }}{{C_{w} - C_{0} }},\quad \omega^{ * } = \frac{d\omega }{{U_{0} }},\quad - \frac{{\partial p^{ * } }}{{\partial x^{ * } }} = \lambda { \exp }\left( {i\omega^{*} t^{*} } \right), \\ \end{aligned}$$

into Eqs. (1)–(4) we get (the “*” symbol is dropped out for the sake of simplicity)

9 $$\phi_{1} Re\frac{\partial u}{\partial t} = \lambda_{0} + \lambda \varepsilon \exp \left( {i\omega t} \right) + \phi_{2} \frac{{\partial^{2} u}}{{\partial y^{2} }} - M^{2} u - \frac{{\phi_{2} }}{k}u + \phi_{3} GrT + \phi_{4} GcC\,,$$

10 $$\left[ {\frac{{\phi_{4} Pe}}{{\lambda_{\text{nf}} }}} \right]\frac{\partial T}{\partial t} = \frac{{\partial^{2} T}}{{\partial y^{2} }} + \frac{{N^{2} }}{{\lambda_{\text{nf}} }}T,$$

11 $$ReSc\frac{\partial C}{\partial t} = \frac{{\partial^{2} C}}{{\partial y^{2} }} + ScSr\frac{{\partial^{2} T}}{{\partial y^{2} }} - Re \gamma ScC,$$

12 $$\begin{array}{*{20}l} {u(0,t) = 0,} \hfill & {u(1,t) = 0,} \hfill \\ {T(0,t) = 0,} \hfill & {T(1,t) = 1,} \hfill \\ {C(0,t) = 0,} \hfill & {C(1,t) = 1,} \hfill \\ \end{array}$$

where

$$\begin{aligned}  Re = \frac{{{\rho}U_{0} d}}{{\mu_{\text{f}} }},\quad M^{2} = \delta \beta_{0}^{2} \frac{{d^{2} }}{{\mu_{{f}} }},\quad Gr = \frac{{g({\beta_{T}})_{\text{f}} d^{2} (T_{w} - T_{0} )}}{{\nu_{\text{f}} U_{0} }},\quad Gc = \frac{{gd^{2}\left( {C_{w} - C_{0} } \right) \left( { \beta_{c} } \right)_{\text{f}} }}{{\nu_{\text{f}} U_{0} }}, \\\,Sr = \frac{{D_{\text{f}} k_{T} (T_{w} - T_{0} ) }}{{\left( {C_{w} - C_{0} } \right)T_{m} \nu_{\text{f}} }},\quad Sc = \frac{{\nu}_{f}}{D_f},\quad {\gamma} = \frac{{k_{r}d}}{{ U_{0} }}, \quad Pe = \frac{{{{ U_{0}d }} \left( \rho c_{p}\right)_{f} }}{{k_{\text{f}} }},\quad N^{2} = \frac{{4\alpha^{2} d^{2} }}{{k_{\text{nf}} }},k = \frac{{k_{1} }}{{d^{2} }},\quad \lambda_{\text{nf}} = \frac{{k_{\text{nf}} }}{{k_{\text{f}} }}, \quad D_{\text{nf}} = \left( 1 - \phi \right) D_{f}. \\ \end{aligned}$$

are Reynold number, Hartmann number, thermal and Solutal Grashof numbers, Soret number, Schmidt number, the Peclet number, radiation and permeability parameters.

After simplification, Eqs. (9)–(11) take the forms:

13 $$a_{o} \frac{\partial u}{\partial t} = \lambda_{0} + \lambda \varepsilon \hbox{e}^{i\omega t} + \phi_{2} \frac{{\partial^{2} u}}{{\partial y^{2} }} - m^{2} u + a_{1} T + a_{2} c,$$

14 $$b_{0}^{2} \frac{\partial T}{\partial t} = \frac{{\partial^{2} T}}{{\partial y^{2} }} + b_{1}^{2} T,$$

15 $$b_{2}^{2} \frac{\partial C}{\partial t} = \frac{{\partial^{2} C}}{{\partial y^{2} }} + b_{3}^{2} \frac{{\partial^{2} T}}{{\partial y^{2} }} - b_{4}^{2} C,$$

where

16 $$\begin{aligned} a_{0} & = \phi_{1} Re,\quad a_{1} = \phi_{3} Gr,\quad a_{2} = \phi_{4} Gc,\quad b_{0}^{2} = \frac{{\phi_{5} Pe}}{{\lambda_{n} }}, \\ \phi_{2} & = \left( {1 + a\phi + b\phi^{2} } \right),\quad b_{1}^{2} = \frac{{N^{2} }}{{\lambda_{n} }},\quad b_{2} = \sqrt {\frac{{ReSc}}{{1-\phi}}} ,\quad b_{3} = \sqrt {ScSr} , \\ b_{4} & = \sqrt {\frac{{Re \gamma Sc}}{{1-\phi}}} ,\quad \phi_{1} = \left( {1 - \phi } \right) + \frac{{\varphi \rho_{\rm s} }}{{\rho_{\text{f}} }},\quad \phi_{3} = \left( {1 - \phi } \right) + \frac{{\phi \left( {\rho \beta_{T} } \right)_{\rm s} }}{{\left( {\rho \beta_{T} } \right)_{\text{f}} }}, \\ \phi_{4} & = \left( {1 - \phi } \right) + \frac{{\phi \left( {\rho \beta_{\text{c}} } \right)_{\text{s}} }}{{\left( {\rho \beta_{\text{c}} } \right)_{\text{f}} }}\quad \phi_{5} = \left( {1 - \phi } \right) + \phi \frac{{\left( {\rho c_{\text{p}} } \right)_{\text{s}} }}{{\left( {\rho c_{\text{p}} } \right)_{\text{f}} }}\,. \\ \end{aligned}$$

In order to solve Eqs. (13)–(15), under boundary condition (12), we suppose the following perturbed type solutions for velocity, temperature and concentration, respectively, as:

17 $$u\,(y,t)\, = \,u_{0} (y) + \varepsilon \,\exp (i\omega t)\,\,u_{1} (y)\,,$$

18 $$T\,(y,t)\, = T_{0} (y)\, + \,\varepsilon \exp \,(i\omega t)T_{1} (y)\,,$$

19 $$C\,(y,t) = \,C_{0} (y) + \varepsilon \exp (i\omega t)C_{1} (y)\,.$$

Using Eqs. (17)–(19) into Eqs. (13)–(15), the below system of ODE’s is obtained:

20 $$\,\,\frac{{\partial^{2} u_{0} (y)}}{{\partial y^{2} }} - m_{1}^{2} u_{0} (y)\, = \, - \lambda_{2} - \frac{{a_{2} \sin b_{1} y}}{{\sin b_{1} }} - a_{3} \left( {\frac{{\sinh b_{4} y}}{{\sinh b_{4} }}\left( {1 + \frac{{b_{1}^{2} b_{3}^{2} }}{{\left( {b_{1}^{2} + b_{4}^{2} } \right)}}} \right) - \frac{{b_{1}^{2} b_{3}^{2} \sin b_{1} y}}{{\left( {b_{1}^{2} + b_{4}^{2} } \right)\sin b_{1} }}} \right)\,,$$

21 $$\frac{{\partial^{2} u_{1} (y)}}{{\partial y^{2} }} - m_{2}^{2} u_{1} (y) = - \lambda_{3} \, ,$$

22 $$\frac{{\partial^{2} T_{0} (y)}}{{\partial y^{2} }} + b_{1}^{2} T_{0} (y) = 0\,,\,$$

23 $$\frac{{\partial^{2} T_{1} (y)}}{{\partial y^{2} }} + m_{3}^{2} T_{1} (y) = 0\,,$$

24 $$\,\,\frac{{\partial^{2} C_{0} }}{{\partial y^{2} }} - b_{4}^{2} C_{0} = b_{1}^{2} b_{3}^{2} \frac{{\sin b_{1} y}}{{\sin b_{1} }}\,\, ,$$

25 $$\,\,\,\,\frac{{\partial^{2} C_{1} }}{{\partial y^{2} }} - b_{5}^{2} C_{1} = 0\,,\,\,$$

where

26 $$a_{2} = \frac{{a_{1} }}{{\phi_{2} }},\quad a_{3} = \frac{{a_{2} }}{{\phi_{2} }},\quad m_{1} = \sqrt {\frac{{m^{2} }}{{\phi_{2} }}} ,\quad m_{2} = \sqrt {\frac{{a_{0} i\omega + m^{2} }}{{\phi_{2} }}} ,\quad \lambda_{2} = \frac{{\lambda_{0} }}{{\phi_{2} }},\quad \lambda_{3} = \frac{{\lambda_{1} }}{{\phi_{2} }}.\,$$

Using Eqs. (17)–(19), the boundary conditions become:

27 $$u_{0} (0) = 0,\quad u_{0} (1) = 0,$$

28 $$u_{1} (0) = 0,\quad u_{1} (1) = 0,$$

29 $$T_{0} (0) = 0,\quad T_{0} (1) = 1,$$

30 $$T_{1} (0) = 0,\quad T_{1} (1) = 0,$$

31 $$C_{0} (0) = 0,\quad C_{0} (1) = 1,$$

32 $$C_{1} (0) = 0,\quad C_{1} (1) = 0.$$

Solutions of Eqs. (22) and (23) using boundary conditions (29) and (30) are:

33 $$T_{0} \left( y \right) = \frac{{\sin b_{1} y}}{{\sin b_{1} }},$$

34 $$T_{1} (y) = 0 .$$

Equation (18), using Eqs. (33) and (34), gives

35 $$T\left( y \right) = \frac{{\sin b_{1} y}}{{\sin b_{1} }}.$$

Equations (24) and (25) under boundary conditions (31) and (32) give

36 $$C_{1} (y,t) = 0,$$

which gives

37 $$C(y,t) = \frac{{\sin hb_{4} y}}{{\sin hb_{4} }}\left( {1 + \frac{{b_{1}^{2} b_{3}^{2} }}{{\left( {b_{1}^{2} + b_{4}^{2} } \right)}}} \right) - \frac{{b_{1}^{2} b_{3}^{2} \sin b_{1} y}}{{\left( {b_{1}^{2} + b_{4}^{2} } \right)\sin b_{1} }}.$$

Equations (21) and (22) result:

38 $$\begin{aligned} u_{0} (y,t) & = c_{1} \sin hm_{1} y + c_{2} \cos hm_{1} y + \frac{{a_{2} \sin b_{1} y}}{{\left( {b_{1}^{2} + m_{1}^{2} } \right)\sin b_{1} }} + \\ & \quad \frac{{a_{3} \sin hb_{4} y}}{{\left( {b_{4}^{2} + m_{1}^{2} } \right)\sin hb_{4} }}\left( {1 + \frac{{b_{1}^{2} b_{3}^{2} }}{{\left( {b_{1}^{2} + b_{4}^{2} } \right)}}} \right) - \frac{{a_{3} b_{1}^{2} b_{3}^{2} \sin b_{1} y}}{{\left( {b_{1}^{2} + m_{1}^{2} } \right)\left( {b_{1}^{2} + b_{4}^{2} } \right)\sin b_{1} }} - \frac{{\lambda_{2} }}{{m_{1}^{2} }}, \\ \end{aligned}$$

39 $$u_{1} \left( {y,t} \right) = c_{3} \sin hm_{2} y + c_{4} \cos hm_{2} y + \frac{{\lambda_{3} }}{{m_{2}^{2} }},$$

with arbitrary constants

40 $$\begin{aligned} c_{1} & = \frac{1}{{\sin hm_{1} }}\left( { - \frac{{\lambda_{2} }}{{m_{1}^{2} }}\cos hm_{1} - \frac{{a_{2} }}{{\left( {b_{1}^{2} + m_{1}^{2} } \right)}} + \frac{{a_{3} b_{3}^{2} b_{1}^{2} }}{{\left( {b_{1}^{2} + m_{1}^{2} } \right)\left( {b_{1}^{2} + b_{4}^{2} } \right)}} - \frac{{a_{3} }}{{\left( {b_{4}^{2} + m_{1}^{2} } \right)}}\left( {1 + \frac{{b_{1}^{2} b_{3}^{2} }}{{\left( {b_{1}^{2} + b_{4}^{2} } \right)}}} \right) + \frac{{\lambda_{2} }}{{m_{1}^{2} }}} \right), \\ c_{2} & = \frac{{\lambda_{2} }}{{m_{1}^{2} }},\quad c_{3} = \frac{{\lambda_{3} \cos hm_{2} }}{{m_{2}^{2} \sin hm_{2} }} - \frac{{\lambda_{3} }}{{m_{2}^{2} \sin hm_{2} }},\quad c_{4} = - \frac{{\lambda_{3} }}{{m_{2}^{2} }}\,. \\ \end{aligned}$$

Finally, substituting Eqs. (38)–(40) into Eq. (17), we get:

41 $$\begin{aligned} u(y,t) & = \left( { - \frac{{\lambda_{2} }}{{m_{1}^{2} }}\cos hm_{1} - \frac{{a_{2} }}{{\left( {b_{1}^{2} + m_{1}^{2} } \right)}} + \frac{{a_{3} b_{3}^{2} b_{1}^{2} }}{{\left( {b_{1}^{2} + m_{1}^{2} } \right)\left( {b_{1}^{2} + b_{4}^{2} } \right)}} - \frac{{a_{3} }}{{\left( {b_{4}^{2} + m_{1}^{2} } \right)}}\left( {1 + \frac{{b_{1}^{2} b_{3}^{2} }}{{\left( {b_{1}^{2} + b_{4}^{2} } \right)}}} \right) + \frac{{\lambda_{2} }}{{m_{1}^{2} }}} \right)\frac{{\sin hm_{1} y}}{{\sin hm_{1} }} \\ & \quad + \frac{{\lambda_{2} }}{{m_{1}^{2} }}\cos hm_{1} y + \frac{{a_{2} \sin b_{1} y}}{{\left( {b_{1}^{2} + m_{1}^{2} } \right)\sin b_{1} }} + \frac{{a_{3} \sin hb_{4} y}}{{\left( {b_{4}^{2} + m_{1}^{2} } \right)\sin hb_{4} }}\left( {1 + \frac{{b_{1}^{2} b_{3}^{2} }}{{\left( {b_{1}^{2} + b_{4}^{2} } \right)}}} \right) - \frac{{a_{3} b_{1}^{2} b_{3}^{2} \sin b_{1} y}}{{\left( {b_{1}^{2} + m_{1}^{2} } \right)\left( {b_{1}^{2} + b_{4}^{2} } \right)\sin b_{1} }}\, \\ & \quad - \frac{{\lambda_{2} }}{{m_{1}^{2} }} + \varepsilon \exp \left( {i\omega t} \right)\left[ {\left( {\frac{{\lambda_{3} \left( {\cosh m_{2} - 1} \right)}}{{m_{2}^{2} \sinh m_{2} }}} \right)\sin hm_{2} y - \frac{{\lambda_{3} }}{{m_{2}^{2} }}\cos hm_{2} y + \frac{{\lambda_{3} }}{{m_{2}^{2} }}} \right]\,. \\ \end{aligned}$$

## Skin Friction and Nusselt Number

The skin friction and heat transfer rate are computed from Eqs. (35) and (41) as follows:

42 $$\begin{aligned} \tau & = \left( { - \frac{{\lambda_{2} }}{{m_{1}^{2} }}\cos hm_{1} - \frac{{a_{2} }}{{\left( {b_{1}^{2} + m_{1}^{2} } \right)}} + \frac{{a_{3} b_{3}^{2} b_{1}^{2} }}{{\left( {b_{1}^{2} + m_{1}^{2} } \right)\left( {b_{1}^{2} + b_{4}^{2} } \right)}} - \frac{{a_{3} }}{{\left( {b_{4}^{2} + m_{1}^{2} } \right)}}\left( {1 + \frac{{b_{1}^{2} b_{3}^{2} }}{{\left( {b_{1}^{2} + b_{4}^{2} } \right)}}} \right) + \frac{{\lambda_{2} }}{{m_{1}^{2} }}} \right)\frac{{m_{1} \cos hm_{1} y}}{{\sin hm_{1} }} \\ & \quad + \frac{{a_{2} b_{1} \cos b_{1} y}}{{\left( {b_{1}^{2} + m_{1}^{2} } \right)\sin b_{1} }} + \frac{{a_{3} b_{4} \cos hb_{4} y}}{{\left( {b_{4}^{2} + m_{1}^{2} } \right)\sin hb_{4} }}\left( {1 + \frac{{b_{1}^{2} b_{3}^{2} }}{{\left( {b_{1}^{2} + b_{4}^{2} } \right)}}} \right) - \frac{{a_{3} b_{1}^{3} b_{3}^{2} \cos b_{1} y}}{{\left( {b_{1}^{2} + m_{1}^{2} } \right)\left( {b_{1}^{2} + b_{4}^{2} } \right)\sin b_{1} }} \\ & \quad + \varepsilon \exp \left( {i\omega t} \right)\left[ {\left( {\frac{{\lambda_{3} \left( {\cos hm_{2} - 1} \right)}}{{m_{2}^{2} \sin hm_{2} }}} \right)m_{2} \cos hm_{2} y} \right], \\ \end{aligned}$$

43 $$Nu = - \frac{{k_{\text{nf}} }}{{k_{\text{f}} }}\left( {\frac{{b_{1} \cos b_{1} }}{{\sin b_{1} }}} \right).$$

## Graphical results and discussion

Heat and mass transfer flow of nanofluids inside a channel is analyzed with radiation impact. Mixed convection MHD, chemical reaction, thermal diffusion with saturated porous medium is taken into account. Cylindrical shaped ${\text{AuNPs}}$ were chosen with kerosene oil as conventional base fluid. Rate of heat transfer is evaluated for various types of nanoparticles, and comparison is made among them. This study mainly focuses on cylindrical shape gold nanoparticles; however, for the sake of comparison, four other types of nanoparticles namely silver, copper, alumina and magnetite are analyzed for the heat transfer rate. Analytical solutions are computed using the perturbation technique and discussed in various plots and tables. The graphical results for velocity (magnetic parameter $M$, permeability parameter $k$, volume fraction $\phi$ and radiation parameter $N$), temperature (for $\phi$ and $N$) and concentration profile (for $\phi$, $N$, Soret number $Sr$ and Reynolds number $Re$) are plotted.

**Fig. 3:**
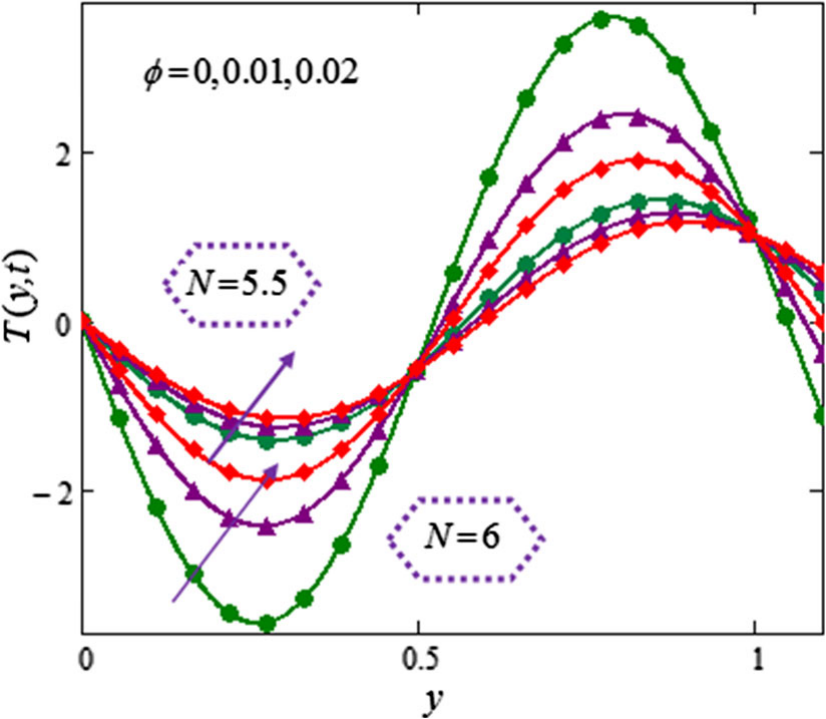
Temperature profile for various values of $\phi$ and $N$ in kerosene oil-based AuNP nanofluid when $t = 1.$

**Fig. 4:**
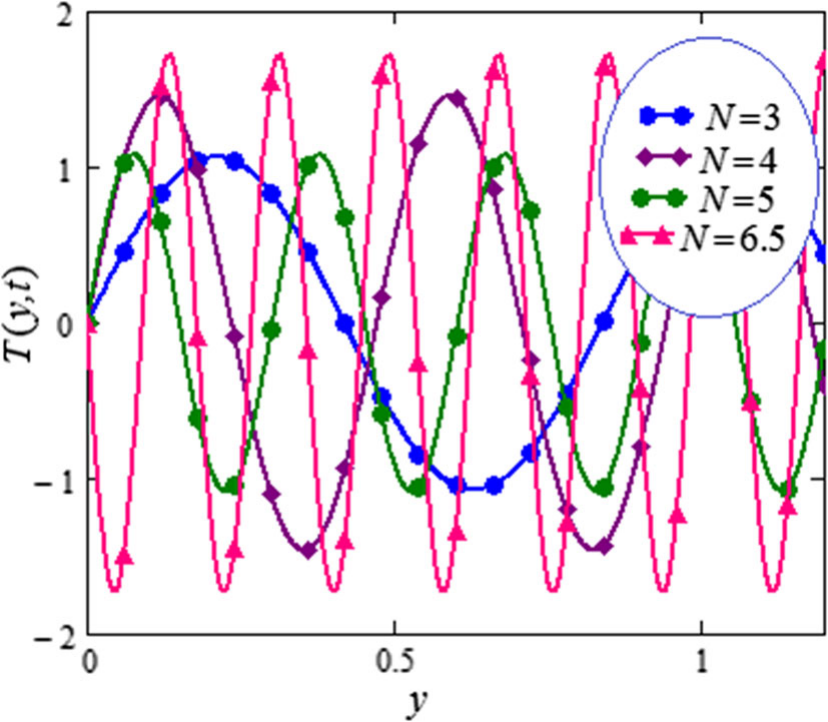
Temperature profile for various values of $N$ in kerosene oil-based AuNP nanofluid when $\,t = 1\,.$

**Fig. 5:**
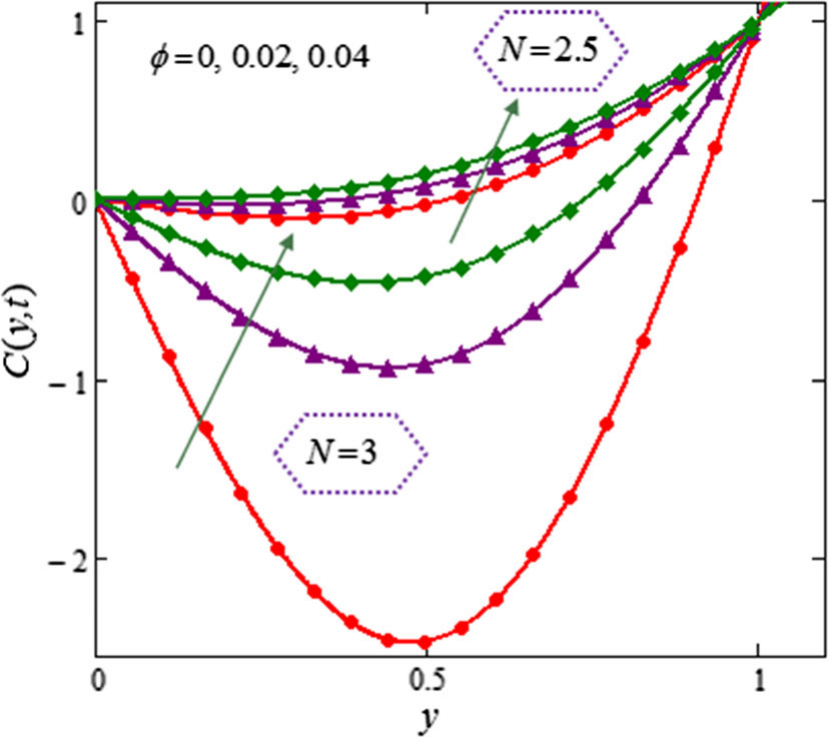
Concentration profile for various values of $\phi$ and $N$ in kerosene oil-based AuNP nanofluid when $\gamma = 2\,,$ $Sr = 0.3,$ $Re = 0.5,$ $Sc = 1.6,$ $t = 2.$

**Fig. 6:**
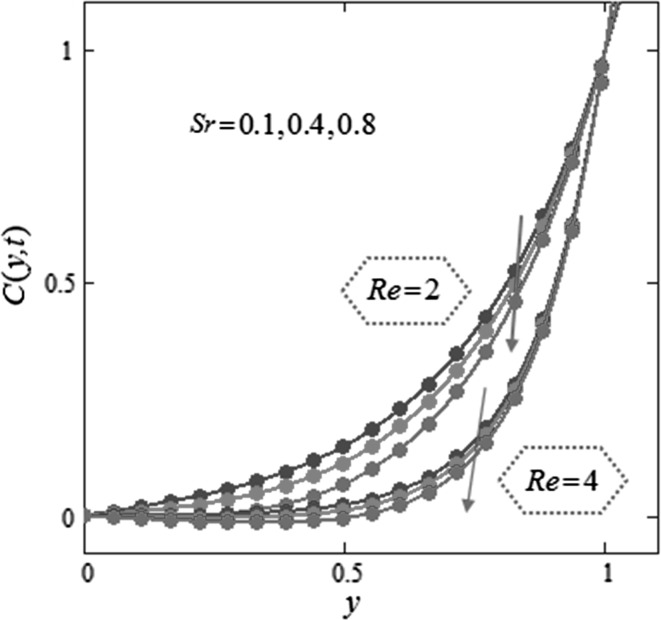
Concentration profile for various values of $Sr$ and $Re$ in kerosene oil-based AuNP nanofluid when $\gamma = 2,$ $N = 1.6,$ $Sc = 1.6,$ $\phi = 0.04,$ $t = 2.$

**Fig. 7:**
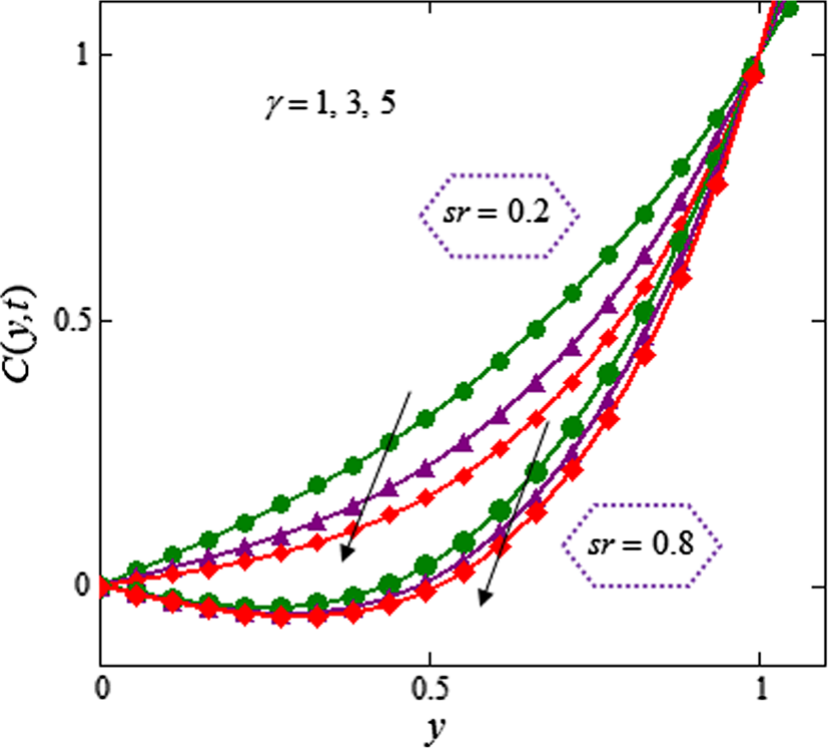
Concentration profile for various values of $\gamma$ and $Sr$ in kerosene oil-based AuNP nanofluid when $N = 2,$ $Sc = 1.6,$ $\phi = 0.03,$ $t = 2,$ $Re = 1.$

**Fig. 8:**
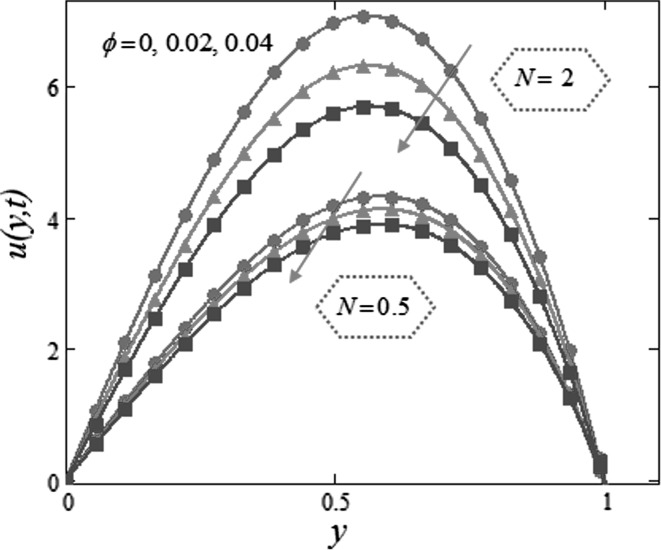
Velocity profile for various values of $\phi$ and $N$ in kerosene oil-based AuNP nanofluid when $Gr = 0.1,$ $Gm = 0.1,$ $Re = 0.3,$ $Sr = 0.7,$ $N = 0.5,$ $M = 1,$ $Sc = 0.5,$ $\varepsilon = 0.5,$ $\lambda = 1,$ $\omega = 0.2,$ $\gamma = 1,$ $k = 1,$ $t = 1\,.$

**Fig. 9:**
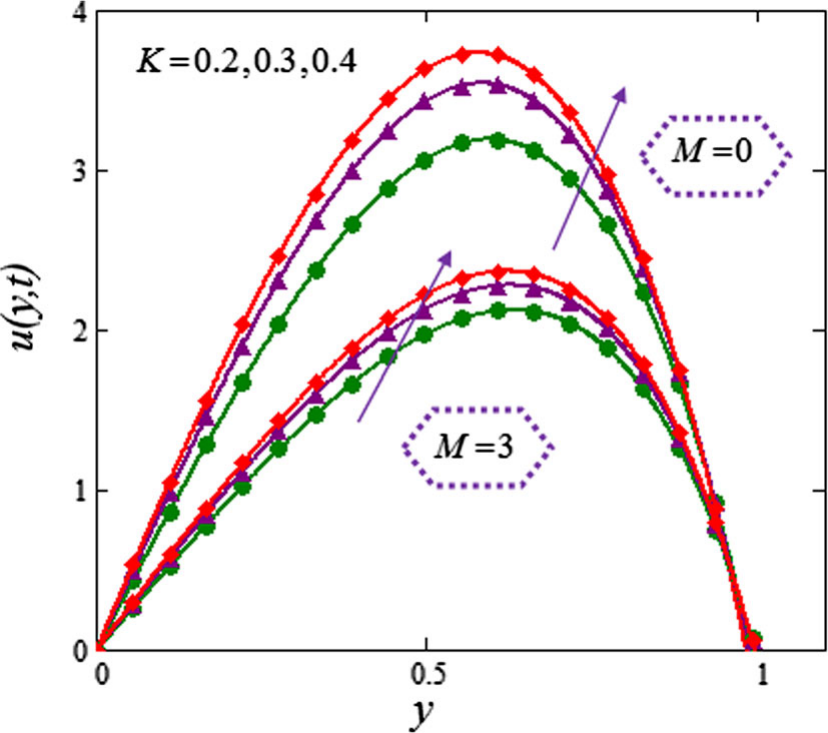
Velocity profile for various values of $k$ and $M$ in kerosene oil-based AuNP nanofluid when $Gr = 0.1,$ $Gm = 0.2,$ $Re = 2,$ $Sr = 0.5,$ $N = 1,$ $Sc = 0.5,$ $\varepsilon = 0.5,$ $\omega = 0.2,$ $\phi = 0.04,$ $\gamma = 0.2,$ $t = 1.$

Figure 3 is mapped to examine the impact of volume fraction $\phi$ along with radiation parameter on nanofluids temperature. It is obvious that sinusoidal effect maximizes with increase in volume fraction $\phi$ and radiation parameter $N.$ Fig. 4 is plotted to show that the temperature profile gets more sinusoidal with increase in $N,$ and this result is in good consent with Aaiza et al. [22]. Figure 5 shows concentration profile for various values of $\phi$ of $AuNP$ and $N$. It is spotted that concentration maximizes with maximizing $\phi$ and suppresses with increasing values of $N$ because of increasing heat energy transferred to the fluid. Figure 6 displays concentration profile for various values of $Sr$ and $Re.$ Concentration profile gets decreased with increasing values of these two parameters. $Sr$ stands for greater temperature gradient, and with greater temperature gradient, concentration profile decreases. Reynolds number $Re$ is actually a ratio of inertial force to viscous force. In this case, inertial forces are dominant than viscous forces; thus, decrease in viscous forces causes a decrease in concentration. Figure 7 shows impact of chemical reaction parameter $\gamma$ and $Sr$ on concentration profile. Concentration exhibits a decrease with increasing values of $\gamma$.The production of energy during chemical reaction causes this fall in concentration profile. Same result was detected by [31]. $Sr$ stands for greater temperature gradient, and with greater temperature gradient, concentration profile suppresses. Figure 8 is plotted to show the effects of $N$ and $\phi$ on velocity of nanofluids. It is detected that velocity profile minimizes with increase in volume fraction $\phi$ of ${\text{AuNP}}$. It is due to the reason that with increasing $\phi$, the fluid gets more viscous. This behavior is identical with that observed by Aaiza et al. [22] and Hamilton and Crosser [31]. Velocity increases with an increase in $N$. Physically, it indicates that by increasing $N$, the amount of heat transfer to the fluid increases. Same output was found by Makinde and Mhone [29]. Figure 9 shows velocity profile for various values of $k$ and magnetic parameter $M.$ It is spotted that velocity maximizes with maximizing values of $k$ owing to smaller friction force. Maximizing $k$ reduces fluid friction with channel wall and fluid flows fast. Velocity suppresses with increasing values of $M$ due to increasing Lorentz forces which opposes the flow of nanofluid. Table 1 shows the empirical shape factors $a$ and $b$ for cylindrical shape nanoparticles. Thermophysical properties of the carrier fluid and different types of nanoparticles are given in Table 2. The sphericity of cylindrical shaped nanoparticles is given in Table 3. Heat transfer rate of nanofluids is evaluated for five different kinds of nanoparticles, i.e., gold, copper, silver, magnetite and alumina with variation in volume fraction $\phi$ of nanoparticles in Table 4. It is observed that nanofluids with cylindrical shaped gold nanoparticles have highest heat transfer rate as compared to metal oxides. A comparison is made between kerosene oil-based metal nanoparticles $({\text{Au}},{\text{Cu}},{\text{Ag}})$ and metal oxide $({\text{Al}}_{2} {\text{O}}_{3} ,{\text{Fe}}_{3} {\text{O}}_{4} )$ nanoparticles. It is detected that metals have highest rate of heat transfer than that of metal oxides because metals have higher thermal conductivities. A comparison is also made between heat transfer rate of regular fluid and nanofluid for different kinds of nanoparticles. It is concluded that nanofluids have higher heat transfer rate as compared to regular fluid, caused by the inclusion of nanoparticles in the fluid. A gradual increase is observed in rate of heat transfer with an increase in $\phi .$ Addition of nanoparticles in the fluid enhances its thermal conductivity which causes an increase in their rate of heat transfer. Gold, silver and copper nanofluids have same rate of heat transfer for different volume fraction, while magnetite and alumina nanofluids have comparatively low heat transfer rate. Moreover, alumina nanofluid has higher heat transfer rate followed by magnetite-based nanofluid because alumina has higher thermal conductivity than magnetite. For all the five nanoparticles, an increase in volume fraction drives a gradual increase in heat transfer rate of nanofluids.

**Table 4:** Influence of volume fraction on heat transfer rate for various kinds of nanoparticles at *N* = 1.5

Volume fraction $\phi$	Gold (Au)	Copper (Cu)	Silver (Ag)	Magnetite $({\text{Fe}}_{3} {\text{O}}_{4} )$	Alumina $({\text{Al}}_{2} {\text{O}}_{3} )$
0	0.106	0.106	0.106	0.106	0.106
0.01	0.156	0.156	0.156	0.153	0.155
0.02	0.201	0.201	0.201	0.195	0.2
0.03	0.243	0.243	0.243	0.235	0.241
0.04	0.28	0.28	0.28	0.271	0.278

## Conclusions

In this paper, impact of cylindrical shaped ${\text{AuNP}}$ on flow of kerosene oil in a vertical channel is investigated. Mixed convection MHD effect is considered along with porous medium. The solution for the governing partial differential equations is evaluated by perturbation technique. The impact of different parameters is observed on velocity, temperature and concentration profiles. Thermal conductivities are found relying on volume fraction of nanoparticles. The deduced observations are:

1. Velocity of nanofluid minimizes with maximization of volume fraction of ${\text{AuNP}}$ owing to amplifying viscosity and thermal conductivity.
2. The drag force increases with increase in magnetic parameter, which slows down velocity of ${\text{AuNP}}$ nanofluid.
3. The velocity of ${\text{AuNP}}$ nanofluid also suppresses with maximizing of Reynolds number.
4. Concentration decreases with maximizing chemical reaction parameter due to emission of heat during chemical reaction.
5. Concentration profile increases with maximizing volume fraction of ${\text{AuNP}}$ and decreases with increase in radiation parameter.
6. Nanofluids with gold nanoparticles have higher rate of heat transfer as compared to metal oxides (alumina and magnetite) due to its high thermal conductivity.
7. Alumina nanofluid has higher heat transfer rate as compared to magnetite-based nanofluid.
8. Heat transfer rate of different types of nanoparticles increases with maximizing volume fraction due to an increase in their thermal conductivities.
